# Protocol for the GAPA person-centred dental care at home intervention: a substudy within a cluster randomised controlled trial in Swedish home care

**DOI:** 10.1186/s12903-026-10340-3

**Published:** 2026-11-03

**Authors:** Jessica Persson Kylén, Sara Björns, Anna Adolfsson, Ulrika Lindmark, Catharina Hägglin, Annica Almståhl, Henrik Imberg, Martin Lundegard, Sofia Torstensson, Elisabeth Söder, Kerstin Karlén, Ann-Christine Baar, Sven Persson Kylén, Anna Bogren, Carina Fjällman, Björn Klinge, Helle Wijk

**Affiliations:** 1 Department of Health Sciences, Karlstad University, Karlstad, 651 88 Sweden; 2 Centre for a DEMentia-friendly Society with A person-centred Mindset, Institute of Health and Care Science, The Sahlgrenska Academy Gothenburg University, Gothenburg, Sweden; 3 Centre for Person-Centred Care, Institute of Health and Care Sciences, Sahlgrenska Academy, University of Gothenburg, Gothenburg, Sweden; 4 Department of Preventive and Community Dentistry, Public Dental Service, Region Västra Götaland, Gothenburg, Sweden; 5 Department of Cariology, Institute of Odontology, Sahlgrenska Academy, University of Gothenburg, Gothenburg, 405 30 Sweden; 6 Department of Oral Medicine and Pathology, Institute of Odontology, Sahlgrenska Academy, University of Gothenburg, Gothenburg, 405 30 Sweden; 7 Department of Research and Development, Public Dental Care, Region Västra Götaland, Gothenburg, Sweden; 8 Department of Behavioural and Community Dentistry, Institute of Odontology, Sahlgrenska Academy, University of Gothenburg, Gothenburg, 405 30 Sweden; 9 Department of Oral Microbiology and Immunology, Institute of Odontology, Sahlgrenska Academy, University of Gothenburg, Gothenburg, 405 30 Sweden; 10 Statistiska Konsultgruppen Sweden AB, Gothenburg, Sweden; 11 Department of Molecular and Clinical Medicine, Institute of Medicine, Sahlgrenska Academy, University of Gothenburg, Gothenburg, Sweden; 12 R&D Department, Primary Health Care, Regionhälsan, Region Västra Götaland, Vänersborg, 462 35 Sweden; 13 Department of Odontology, Umeå University, Umeå, 901 87 Sweden; 14 Department of Health Sciences, University West, Trollhättan, 461 86 Sweden; 15 Department of Periodontology, Faculty of Odontology, Malmö University, Malmö, Sweden; 16 Department of Dental Medicine, Division of Oral Diseases, Karolinska Institutet, Stockholm, Sweden; 17 Institute of Health and Care Sciences, Sahlgrenska Academy, Gothenburg University, Box 457, Gothenburg, 405 30 Sweden; 18 Quality and Patient Safety Unit, Sahlgrenska University Hospital of Gothenburg, Gothenburg, 413 45 Sweden; 19 Centre for Healthcare Architecture, CVA, Chalmers University of Technology, Gothenburg, 412 96 Sweden

**Keywords:** Bedside dental care, Home care, Older adults, Online dentistry, Oral health

## Abstract

**Background:**

Oral health is essential to healthy ageing and closely linked to well-being, social participation, and quality of life. Frail older adults are at high risk of oral diseases, yet oral health is often insufficiently integrated into home care. Care staff frequently report limited competence and unclear routines, while older adults wish to participate actively in decisions about their oral health. GAPA Person-Centered Dental Care @ Home (Dental PCC@Home) is a co-designed model developed with older adults, significant others (informal caregivers), municipal care staff, and dental professionals. This substudy evaluates if Dental PCC@Home will promote oral health, participation, and self-determination among older adults living with complex care needs.

**Methods:**

Dental PCC@Home is embedded within the parent GAPA cluster randomised controlled trial. Decisions about dental care are guided by a patient decision aid and discussion of needs expressed by the older adult or identified by care staff. Participation in the substudy is voluntary. Eligible intervention-group participants are offered a patient decision aid to support decisions about whether and how to receive dental care, followed by one of four pathways: online dental consultation, mobile home dental care, a dental clinic visit, or declining dental care. Eligible control-group participants receive standard care. Oral health and quality-of-life assessments are conducted at baseline and at six and twelve months; cognitive function is assessed at baseline only. Co-primary outcomes are changes in oral health status (ROAG) and oral health-related quality of life (GOHAI) from baseline to twelve months. General health-related quality of life (EQ-5D-5 L) is a secondary outcome. Quantitative analyses will account for clustering and repeated measurements. Semi-structured interviews with older adults, significant others, and care staff will explore experiences of care, decision-making, and implementation and will be analysed using qualitative content analysis.

**Discussion:**

This project will generate evidence on a new cross-organisational model designed to improve access to oral health care for frail older adults living at home. By integrating dental care into existing care processes and offering flexible care pathways, the model has the potential to strengthen autonomy, reduce inequalities, promote health and, when indicated, address oral diseases earlier in home care. The findings may inform future practice, policy development, and person-centred approaches to oral health in ageing populations.

**Trial registration:**

ClinicalTrials.gov NCT06310798. Registered on March 13, 2024.

**Supplementary Information:**

The online version contains supplementary material available at https://doi.org/10.1186/s12903-026-10340-3.

## Introduction

Oral health is a basic human right essential to healthy ageing [1, 2]. The ability to eat, speak, smile, and take part in social activities without pain is vital for well-being and self-image [3]. Frail older adults have a substantially increased risk of oral diseases [4, 5]. Untreated oral conditions in this population are associated with pain, infection and impaired nutrition, highlighting the need for systematic oral health assessment [6]. Management within home care should incorporate regular oral health checks into nursing practice and strengthen collaboration with oral health professionals. Moreover, oral health is a key component of quality of life in old age [7].

As we grow older, the risk of losing our regular dental contact is high [8]. With the current trend towards growing numbers of older adults with complex care needs living at home, the need to integrate oral health care tasks into daily care increases. Care staff frequently express lacking competence on oral health, and that routines, attitudes and responsibilities around oral care are unclear [9–13]. Older adults want to be involved in decisions regarding their oral health [14]. Providing oral health care involves not only delivering assistance with care tasks but also enabling participation and respecting autonomy. Person-centred care means seeing the older adult as a partner in care, where their values, preferences and everyday life will guide decisions [15]. It shifts the focus from doing things for the older adult to doing things with them, ensuring dignity and meaningful involvement. Balancing older adults’ right to autonomy with the care system’s role of identifying, and responding to needs respectfully, can be a complex task for the individual care provider [14]. Here we identify an urgent need to develop shared ways of working with older adults, providers of care, and medical and dental professionals that strengthen participation and clarify roles in care processes.

For the past 25 years, Swedish older adults with extensive care needs have had a legal right to a national dental remuneration programme [16]. The programme provides highly subsidised dental care; at the same low cost as healthcare visits, together with an annual oral health screening at the home of each older adult. A licensed dental professional (mainly dental hygienists) carries out the screening and documents the findings and recommendations on a paper document, an oral care card. Care staff (assistant nurses) are encouraged to attend the oral health screenings.

In our ongoing cluster randomised controlled trial (GAPA cRCT), we evaluate a new cross-organisational work model (GAPA, in Swedish ‘Gemensam Arbetsmodell för Personcentrerad sAmverkan’. In English ‘Work-model for person-centred oral health care planning in home care’) developed in response to previously identified barriers to oral health care for older adults with extensive care needs [9–13]. The model is grounded in the existing dental remuneration programme and involves collaboration between dental professionals and care staff to conduct oral health assessments in the homes of frail older adults [16].

Through co-creation new working methods have been developed together with end-users that are expected to better meet the actual needs expressed by older adults and staff [17]. For example, in a pilot study involving older adults receiving home healthcare and nurses from seven municipalities, the older adults clearly expressed a wish for dental care at home, as their fragile health made visits to clinics difficult or impossible [14]. The nurses highlighted the importance of home dental care from their point of view, since they lacked someone to consult about oral health in daily practice. This reveals a structural gap between older adults’ needs and the availability of care, where current systems fail to meet the needs of the most fragile. Home dental care is a priority in Swedish national guidelines for dental care, but scientifically evaluated working models are missing. 

‘GAPA Person-Centred Dental Care @ Home’ (Dental PCC@Home**)**, is a substudy within the overarching GAPA cRCT [18]. It is pioneering in combining research, innovation, and practice through co-creation based on lived experience. By integrating dental care into a continuous care process, participation, equality and autonomy could potentially be promoted, especially regarding groups at risk of being excluded from traditional care structures. The cross-organisational infrastructure established within the wider GAPA project enables research results to quickly be translated into practice. Dental PCC@Home is thus not a stand-alone effort, but a strategic part of improving person-centred care.

The aim of this substudy is to evaluate whether Dental PCC@Home promotes oral health, participation, and self-determination among older adults with complex care needs through home-based dental care, integrated within a cross-organisational care process.

- How do older adults with complex care-needs experience dental treatment at home?
- How can oral health and dental care be more integrated into home care processes?
- What facilitators and barriers arise in a coherent, cross-organisational care process within Dental PCC@Home?
- What is feasible within Dental PCC@Home from an odontological, oral health, health-promoting and occupational health perspective?

## Methods

### Study design and setting

This substudy protocol should be read alongside the published protocol for the parent GAPA cluster randomised controlled trial [18]. Unless otherwise specified, the substudy follows the procedures described in the parent protocol. The present protocol details the Dental PCC@Home intervention and substudy-specific procedures.

**Fig. 1:**
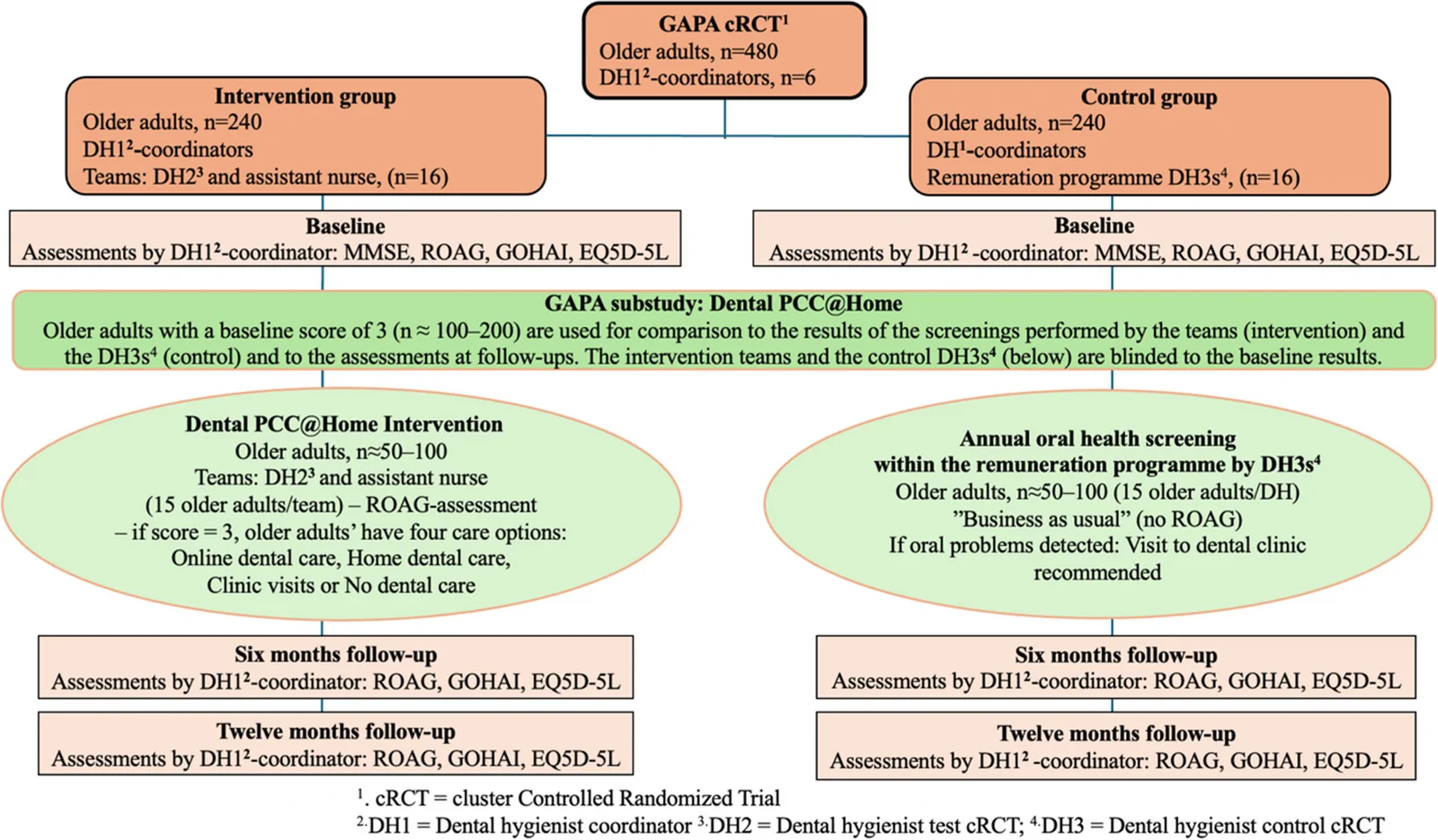
Evaluation process for the GAPA Person-Centred Dental Care at Home (Dental PCC@Home) study

Dental PCC@Home will be implemented as a defined intervention within a larger cluster-randomised controlled trial (GAPA cRCT). The overall study design has been published previously in a separate study protocol [18]. In brief, the GAPA cRCT will include a total of 480 older adults enrolled within the dental care remuneration programme and home care (240 in the intervention group and 240 in the control group = business as usual) across 14 municipalities in the west of Sweden. Randomisation will be performed at the municipality level. The trial will involve 32 dental hygienists, each responsible for approximately 15 older adults. The overall study design and data collection procedures of the Dental PCC@Home substudy are illustrated in Fig. 1.

As illustrated in Fig. 1, Dental PCC@Home is embedded within the parent GAPA cRCT. Assessments are conducted by six trained coordinators/dental hygienists at baseline and at six and twelve months. Participation in the Dental PCC@Home substudy is discussed with the older adult in relation to needs expressed by the person or identified by care staff, using the patient decision aid [19]. Eligible intervention-group participants are offered the Dental PCC@Home care pathways, while eligible control-group participants receive standard care. There is no additional randomisation for the substudy.

For evaluation the following validated instruments will be used:

- Mini Mental State Examination (MMSE) [20]: an instrument designed to assess cognitive function and potential cognitive impairment. Cognitive function is assessed at baseline only.
- Revised Oral Assessment Guide (ROAG) [21]: is a standardized clinical instrument designed for use by nurses and care staff to support systematic assessment of oral health status, identification of risks, and guidance for appropriate interventions or referral to dental services in frail older adults.
- General Oral Health Assessment Index (GOHAI) [22, 23]: is a validated instrument for evaluating oral health-related quality of life. It focuses on functional limitation, pain or discomfort, and psychosocial impacts related to oral health.
- EQ-5D-5 L [24]: is a standardized, generic instrument for assessing health-related quality of life. It comprises five dimensions (mobility, self-care, usual activities, pain/discomfort, and anxiety/depression).

### Participants and eligibility criteria

Within the GAPA cRCT, assistant nurses and dental hygienists ask older adults whether they wish to participate in the Dental PCC@Home substudy. Discussion of dental care is guided by the patient decision aid developed within the project [19], taking into account needs expressed by the older adult and needs identified by care staff. Participation is voluntary; older adults may decline participation or withdraw at any time.

### Intervention: GAPA person-centred dental care at home

Participants included in the substudy and allocated to the intervention group will be offered Dental PCC@Home pathways following oral health assessment and shared decision-making. A patient decision aid will be used to support decisions regarding whether dental care is desired and, if so, in what form. The decision aid has been developed [19] based on a prior decisional needs assessment [14], informed by the Ottawa Decision Support Framework [15], and guided by the International Patient Decision Aid Standards (IPDAS) [25]. Four alternative dental care pathways will be offered: online dental consultation, home-based dental care, clinic-based dental care, or the option to decline dental care.

- Online dental consultation: Municipal care staff and dental hygienists use headsets to connect with a remote dentist at the digital dental clinic within the Public Dental Service. The dentist participates remotely in a consultation with the older adult, assistant nurse, and dental hygienist in the older adult’s home. This pathway supports discussion of oral health findings and possible care needs following oral health assessment and planning within the parent GAPA cRCT. The consultation is offered free of charge to the older adult.
- Home dental care: The second option is dental care at home, provided through mobile home dental care, where a dentist and a dental nurse make home visits and offer suitable indicated dental treatments on site. Basic dental care, such as to remove and polish sharp edges, or to provide a temporary filling for caries, will be available to the older adult at healthcare fee (200 SEK, approximately 20 Euro; in line with the Swedish Dental care remuneration programme [16]). For older adults with high-cost protection, or those aged over 85, this will be free of charge.
- Clinic visits: Within the project, dental care at a clinic will be offered to those older adults who request it. The dental hygienist in the team is responsible for booking the older person at the clinic where the older adults wish to receive dental care, within the fee for dental care remuneration programme (maximum 200 SEK). For those with high-cost protection, or aged over 85, dental care is free of charge.
- No dental care: For those older adults who do not wish to have any dental care, this is an option. Regardless their decision, they will receive information with assistance of the patient decision aid about how their choice might affect their overall health and oral health.

### Control group

Participants included in the substudy and allocated to the control group will receive standard care, i.e., business as usual. Standard care will consist of clinic-based dental care or no dental care for participants who decline such services, in accordance with usual practice and existing routines within municipal and dental care services. The dental hygienists performing the oral assessments in the control group will recommend older adults to visit the dental care clinic if this is indicated.

### Data collection

Oral health status, oral health-related quality of life, and general health-related quality of life are assessed at baseline and at six- and twelve-month follow-up by six trained coordinators/dental hygienists. Cognitive function is assessed using the MMSE at baseline only. The instruments are described under Study design and setting. Dental hygienists involved in data collection undergo a two-day calibration programme to support standardised assessments.

### Outcomes

The Dental PCC@Home substudy will evaluate outcomes consistent with those defined in the GAPA cRCT. The primary outcomes will be older adults’ oral health status and oral health-related quality of life, assessed using the Revised Oral Assessment Guide (ROAG; 19) and the Geriatric Oral Health Assessment Index (GOHAI; 21, 22), respectively. The main secondary outcome will be general health-related quality of life, measured using the EQ-5D-5 L (23). Additional secondary outcomes related to participation, shared decision-making, and healthcare utilisation will be evaluated in line with the GAPA cRCT trial protocol [18]. Within the Dental PCC@Home substudy, supplementary descriptive analyses will describe uptake of dental care, selected care pathways, and patterns of dental care use among participants meeting the substudy inclusion criterion.

### Qualitative data collection and analysis

Qualitative data collection includes semi-structured interviews with older adults, family members or informal carers, and care staff. All older adults who received dental care at home were interviewed immediately after receiving care. These interviews were conducted by JPK and ST, who assisted the dentist. Interviews address experiences of oral health assessment, decision-making, care pathways, and perceived facilitators and barriers to person-centred dental care at home. Interview data will be analysed using qualitative content analysis, following an inductive approach to identify meaning units, codes, subcategories, and overarching categories [26, 27].

### Statistical analysis

Statistical analyses for the Dental PCC@Home substudy will follow the analytical principles specified in the GAPA cRCT. All efficacy analyses will be conducted according to the intention-to-treat principle, including all eligible participants according to their randomised allocation.

Primary analyses will evaluate changes in oral health status (ROAG; 19) and oral health-related quality of life (GOHAI; 21,22) from baseline to 12-month follow-up, comparing intervention and control groups. Secondary analyses will assess general health-related quality of life (EQ-5D-5 L; 23) and other secondary outcomes defined in the GAPA cRCT protocol. Longitudinal analyses will be performed using appropriate mixed-effects regression models to account for the clustered trial design, with dental hygienist included as a random effect. Models will be adjusted for baseline values where applicable. The two primary endpoints, change in ROAG [21] and GOHAI [22, 23] from baseline to 12-month follow-up, will be tested at a two-sided significance level of α = 0.025, thereby maintaining an overall type I error rate of 5% for the co-primary endpoints.

Additional exploratory analyses will be conducted to describe uptake of dental care, selected care pathways, and patterns of healthcare utilisation within the substudy population. These analyses will be descriptive in nature. All statistical tests will be two-sided and conducted at a significance level of 5%, and effect estimates will be presented with 95% confidence intervals, unless otherwise stated. No formal adjustment for multiple comparisons will be applied for secondary or exploratory analyses.

### Sample size

Based on previous research, approximately 20–40% of participants in the GAPA cRCT were initially expected to require dental care [14], corresponding to an estimated 50–100 participants per group (approximately 100–200 participants in total). Under the assumptions of the parent trial [18], a substudy sample size of 100 participants per group would allow detection of a mean difference in ROAG of approximately 1.5 units (standardised effect size 0.5), while a sample size of 50 participants per group would allow detection of a mean difference in ROAG of approximately 2 units (standardised effect size 0.67) at 80% power. As eligibility for the substudy is determined at the participant level, the distribution of dental hygienists and municipalities in the substudy is expected to be similar to that of the overall trial. Sample size calculations were performed using SAS/STAT^®^ software (version 9.4; SAS Institute Inc, Cary, NC, USA) POWER procedure, with adjustment for clustering as appropriate for cluster-randomised trials [28].

### Trial status

Data collection for the Dental PCC@Home substudy is ongoing. The six-month follow-up of the parent GAPA cluster randomised controlled trial was completed as planned during August–September 2026. A total of 23 older adults has currently received dental care at home within the substudy. The twelve-month follow-up is planned. This status reflects information available on 7 October 2026.

## Discussion

Dental PCC@Home has been shaped through several years of co-creation involving older adults, their significant others, and the staff working closest to them. Organisational aspects have also been addressed continuously through collaborative projects. Dental PCC@Home now needs to be evaluated using scientifically validated methods.

This research addresses existing gaps in knowledge, and its significance and contribution can be summarised in the following fields:

### Person-centred care, participation and autonomy

- There is a clear need for research regarding the understanding of how person-centred oral health care can be operationalised in everyday practice, particularly for older adults with complex needs [29–31]. Current evidence offers limited guidance on how participation, autonomy and shared decision-making can be supported across organisational boundaries [14]. Through Dental PCC@Home, we are uniquely positioned to generate this knowledge by evaluating a model that strengthen person-centred care in real-world settings.
- Dental PCC@Home enables treatment despite limited mobility and/or cognitive impairment, for example older adults living with dementia, thereby strengthening self-determination and the sense of control for older adults with frailty in line with a gap in research [29–31].
- Even though oral health is closely linked to autonomy and older adults in home care are at high risk for oral diseases, there seems to be no patient decision aids developed for assisting older adults in making decisions about their oral health [32]. A decision aid for frail older adults has been developed during this project [19]. It will be tested during this trial. Hopefully it will support older adults in making evidence-based decisions that are anchored in what matters most to them.

### Integration, organisation and care pathways

- Oral health is not fully integrated into care processes in municipal care for frail older adults [9–13]. This leads to unequal access to care where actions are based on if you have significant others that might give voice to your concerns. Within Dental PCC@Home a new care process is tested that both strengthens prevention and creates a coherent oral care pathway for older adults living with complex care needs regardless of their context. This may facilitate more equal, integrated care.
- Care staff across different organisations (municipal services and dental care) establish an infrastructure for oral health care planning that is currently lacking [6, 30, 31, 33], where eventually oral diseases are treated in the home of the older adult. This is expected to save resources and energy in the form of reduced travel for older adults and their families. However, the literature in this area is limited and a health economic evaluation will be conducted as part of the project.

### Health outcomes, prevention and quality of life

- Oral diseases are addressed in Dental PCC@Home, hopefully reducing suffering, and worsening of other conditions, which may improve quality of life. Good oral health can support improved nutritional status and social functioning, and also limit the burden of oral infection/inflammation on systemic health [34, 35]. Significant others will also be offered the opportunity to be included in planning and follow-up, which has been identified as a need in previous research within this project [36].
- Among older adults living in long-term care facilities in Sweden, approximately 70% have been found to suffer from caries disease [10], leading to pain, reduced quality of life and increasing care needs. Dental PCC@Home adopts a health-promoting and preventive approach, aiming through cross-organisational collaboration to reach older adults before they move into long-term care, that is, during the period when they receive home care and/or home healthcare.

### Equity, access and vulnerable populations

- Dental PCC@Home addresses a concrete need among a vulnerable group who often fall outside regular dental care by losing their previous routine dental contact [8]. By offering home-based dental care tailored to the older adult’s needs and preferences, equality, quality of life and preventive health are promoted.

### Digital innovation and technology-enabled care

- To our notion, the evaluation of online consultation through VR is a new method in dentistry. It has the potential to improve quality of care by combining digital technology with person-centred care. Older adults who do not wish to or are unable to travel to a dental clinic can consult a dentist about possible oral disease, which is expected to save resources, time and energy for older adults, significant others and staff across organisations.
- Older adults, significant others, and care staff may have differing levels of comfort and familiarity with digital technologies [13, 14, 36]. These differences could shape willingness to engage in online consultations, affect perceived participation, and influence the acceptability of the digital components of the intervention. Qualitative data will help clarify for whom and under what circumstances digital dental care is experienced as meaningful.

## Supplementary Information

Supplementary Material 1.

## Data Availability

The datasets generated during the present study are not publicly available due to ethical and legal restrictions; however, access may be considered upon reasonable request and subject to ethical approval, in accordance with the GDPR and applicable local legislation.
