# Supplementary material for: Protocol for the GAPA person-centred dental care at home intervention: a substudy within a cluster randomised controlled trial in Swedish home care
Source: BMC Oral Health. 2026 Nov 3;26:2173. doi: 10.1186/s12903-026-10340-3 (PMC13733002; doi:10.1186/s12903-026-10340-3)
Supplement: Supplementary file 1 [file 12903_2026_10340_MOESM1_ESM.docx]

SPIRIT 2025 checklist for Dental PCC at Home

Manuscript: Protocol for the GAPA Person-Centred Dental Care at Home Intervention: A Substudy within a Cluster Randomised Controlled Trial in Swedish Home Care.

Completed against the revised Dental PCC@Home substudy manuscript and the published parent GAPA cRCT protocol (reference 18), supplied by the authors. Substudy locations are given as section names; parent-protocol locations use its printed page numbers (13-page article). “Not reported” means information was not identified in either document. Where reporting is partial, the remaining limitation is specified.

Source: Chan A-W et al. SPIRIT 2025 statement. PLoS Medicine. 2025;22:e1004589. doi:10.1371/journal.pmed.1004589. Checklist descriptions reproduced from the SPIRIT 2025 statement under the Creative Commons Attribution licence.

| **Item** | **SPIRIT 2025 item description** | **Manuscript location and reporting note** |
| --- | --- | --- |
| 1a | Title stating the trial design, population, and interventions, with identification as a protocol | Substudy: Title Title identifies the intervention, home-care population, cluster randomised design, and protocol. |
| 1b | Structured summary of trial design and methods, including items from the World Health Organization Trial Registration Data Set | Substudy: Abstract Structured abstract; registration stated. Not all WHO registration dataset fields are included. |
| 2 | Version date and identifier | Parent protocol p. 11, Trial status: first protocol dated 25 November 2024. Substudy: Trial status dated 7 October 2026, but no separate version identifier. |
| 3a | Names, affiliations, and roles of protocol contributors | Substudy: Title page and Authors’ contributions Author names, affiliations, corresponding author, and contributions. |
| 3b | Name and contact information for the trial sponsor | Substudy: Title page Corresponding-author contact is supplied, but trial sponsor and sponsor contact are not explicitly identified. |
| 3c | Role of trial sponsor and funders in design, conduct, analysis, and reporting of trial; including any authority over these activities | Substudy: Funding Funders are named; their roles and authority are not specified. |
| 3d | Composition, roles, and responsibilities of the coordinating site, steering committee, endpoint adjudication committee, data management team, and other individuals or groups overseeing the trial, if applicable | Parent protocol pp. 6, 9–10, Phase V and Phase VII: steering committee, project management group, six coordinators, oversight roles and meeting frequency specified. |
| 4 | Name of trial registry, identifying number (with URL), and date of registration. If not yet registered, name of intended registry | Substudy: Trial registration ClinicalTrials.gov NCT06310798; registered 13 March 2024. Registry URL is not given in the manuscript. |
| 5 | Where the trial protocol and statistical analysis plan can be accessed | Substudy: Study design and setting and Statistical analysis, reference 18. Parent published protocol available at doi:10.1186/s13063-025-08753-6. Separate statistical analysis plan access not specified. |
| 6 | Where and how the individual de-identified participant data (including data dictionary), statistical code, and any other materials will be accessible | Substudy: Availability of data and materials Restricted data access on reasonable request subject to ethical approval and GDPR. Data dictionary and statistical code access are not specified. |
| 7a | Sources of funding and other support (eg, supply of drugs) | Substudy: Funding Funding sources and grant numbers stated; external funder peer review explicitly confirmed. |
| 7b | Financial and other conflicts of interest for principal investigators and steering committee members | Substudy: Competing interests Authors declare no competing interests. Steering committee conflicts are not separately addressed. |
| 8 | Plans to communicate trial results to participants, healthcare professionals, the public, and other relevant groups (eg, reporting in trial registry, plain language summary, publication) | Substudy: Discussion. |
| 9a | Scientific background and rationale, including summary of relevant studies (published and unpublished) examining benefits and harms for each intervention | Substudy: Introduction Background, prior research, rationale, and co-development described. Potential harms are not systematically reviewed. |
| 9b | Explanation for choice of comparator | Substudy: Introduction and Control group Existing remuneration programme and standard care described. |
| 10 | Specific objectives related to benefits and harms | Substudy: Aim and research questions Aim and research questions reported. |
| 11 | Details of, or plans for, patient or public involvement in the design, conduct, and reporting of the trial | Parent protocol pp. 4–6, development phases, and pp. 9–10, Phase VII and Discussion: co-development, patient representation on steering committee, and older-adult involvement including authorship. Substudy: Introduction and Discussion. |
| 12 | Description of trial design including type of trial (eg, parallel group, crossover), allocation ratio, and framework (eg, superiority, equivalence, non-inferiority, exploratory) | Substudy: Study design and setting Substudy within municipality-randomised parent cRCT; planned parent groups of 240 each. |
| 13 | Settings (eg, community, hospital) and locations (eg, countries, sites) where the trial will be conducted | Substudy: Study design and setting Home care across 14 municipalities in western Sweden; planned parent-trial setting reported. |
| 14a | Eligibility criteria for participants | Substudy: Participants and eligibility criteria. Parent protocol p. 8, Phase VI: home care and dental remuneration programme; exclusion below age 65. Substudy participation follows needs discussion and voluntary invitation. |
| 14b | If applicable, eligibility criteria for sites and for individuals who will deliver the interventions (eg, surgeons, physiotherapists) | Parent protocol p. 8, Phase VI: dental hygienists conducting remuneration-programme assessments and nursing assistants working in home care. Substudy: Intervention and Data collection. |
| 15a | Intervention and comparator with sufficient details to allow replication including how, when, and by whom they will be administered. If relevant, where additional materials describing the intervention and comparator (eg, intervention manual) can be accessed | Substudy: Intervention and Control group. Parent protocol pp. 6–8, Phase VI: team model, assessment tools, digital platform, provider training and standard care. Substudy-specific pathways take precedence over parent pathway descriptions. |
| 15b | Criteria for discontinuing or modifying allocated intervention/comparator for a trial participant (eg, drug dose change in response to harms, participant request, or improving/worsening disease) | Parent protocol p. 9, Safety: no special discontinuation or modification criteria. Substudy: Participants and eligibility criteria and Intervention: voluntary participation, withdrawal and declining dental care. |
| 15c | Strategies to improve adherence to intervention/comparator protocols, if applicable, and any procedures for monitoring adherence (eg, drug tablet return, sessions attended) | Substudy: Intervention |
| 15d | Concomitant care that is permitted or prohibited during the trial | Substudy: Intervention and Control group |
| 16 | Primary and secondary outcomes, including the specific measurement variable (eg, systolic blood pressure), analysis metric (eg, change from baseline, final value, time to event), method of aggregation (eg, median, proportion), and time point for each outcome | Substudy: Outcomes and Statistical analysis ROAG, GOHAI, EQ-5D-5L; baseline-to-twelve-month change for co-primary outcomes. |
| 17 | How harms are defined and will be assessed (eg, systematically, non-systematically) | Parent protocol pp. 8–9, Phase VI and Safety: coordinators manage spontaneous reports; adverse events recorded at six and twelve months with severity, duration and causality. Substudy: Ethics approval and consent to participate adopts parent procedures. |
| 18 | Time schedule of enrolment, interventions (including any run-ins and washouts), assessments, and visits for participants. A schematic diagram is highly recommended (see fig 1 ) | Substudy: Figure 1, Data collection and Trial status Figure 1 and baseline/six-/twelve-month assessments; MMSE at baseline only. Figure 1 still contains a ROAG eligibility criterion inconsistent with revised eligibility text. |
| 19 | How sample size was determined, including all assumptions supporting the sample size calculation | Substudy: Sample size. Parent protocol p. 9, Data analysis: SD 3, baseline/change correlation 0.3, 15 older adults per DH, ICC 0.05, alpha 0.025, 25% dropout and 80% power. These are parent-trial assumptions. |
| 20 | Strategies for achieving adequate participant enrolment to reach target sample size | Substudy: Participants and eligibility criteria. Parent protocol pp. 7–8, Phase VI: remuneration-programme population and planned 15 older adults per team/DH. |
| 21a | Who will generate the random allocation sequence and the method used | Parent protocol p. 8, Phase VI: dental hygienists randomised in pairs; generator and method not specified. Substudy: Study design and setting describes municipality-level randomisation. The stated randomisation unit differs between documents. |
| 21b | Type of randomisation (simple or restricted) and details of any factors for stratification. To reduce predictability of a random sequence, other details of any planned restriction (eg, blocking) should be provided in a separate document that is unavailable to those who enrol participants or assign interventions | Parent protocol p. 8, Phase VI: randomisation in DH pairs and geographically linked nursing assistants described; restrictions, stratification and sequence details not fully specified. |
| 22 | Mechanism used to implement the random allocation sequence (eg, central computer/telephone; sequentially numbered, opaque, sealed containers), describing any steps to conceal the sequence until interventions are assigned | Substudy: Study design and setting Allocation concealment mechanism not specified in this manuscript. |
| 23 | Whether the personnel who will enrol and those who will assign participants to the interventions will have access to the random allocation sequence | Substudy: Study design and setting Access to the allocation sequence by recruiting/assigning personnel not specified. |
| 24a | Who will be blinded after assignment to interventions (eg, participants, care providers, outcome assessors, data analysts) | Substudy: Methods Blinding status is not stated. |
| 24b | If blinded, how blinding will be achieved and description of the similarity of interventions | Substudy: Methods Blinding methods not stated; applicability cannot be determined from manuscript. |
| 24c | If blinded, circumstances under which unblinding is permissible, and procedure for revealing a participant’s allocated intervention during the trial | Substudy: Methods Unblinding procedures not stated; applicability cannot be determined from manuscript. |
| 25a | Plans for assessment and collection of trial data, including any related processes to promote data quality (eg, duplicate measurements, training of assessors) and a description of trial instruments (eg, questionnaires, laboratory tests) along with their reliability and validity, if known. Reference to where data collection forms can be accessed, if not in the protocol | Substudy: Study design and setting, Data collection, Qualitative data collection and analysis. Parent protocol pp. 7–9, Figure 3 and Phase VI: baseline/follow-up instruments, assessment training and retrospective records review. |
| 25b | Plans to promote participant retention and complete follow-up, including list of any outcome data to be collected for participants who discontinue or deviate from intervention protocols | Substudy: Participants and eligibility criteria and Data collection Withdrawal permitted and follow-up schedule stated; retention strategy and data collection after withdrawal not specified. |
| 26 | Plans for data entry, coding, security, and storage, including any related processes to promote data quality (eg, double data entry; range checks for data values). Reference to where details of data management procedures can be accessed, if not in the protocol | Substudy: Ethics approval and consent to participate and Availability of data and materials. Parent protocol p. 11, Data availability: permission for study use and GDPR restrictions. Operational entry, coding, security, storage and quality-check procedures not specified. |
| 27a | Statistical methods used to compare groups for primary and secondary outcomes, including harms | Substudy: Statistical analysis. Parent protocol p. 9, Data analysis: DH random effect, baseline adjustment, effect estimates and significance levels. Harms analysis not explicitly specified; randomisation-unit difference between documents remains. |
| 27b | Definition of who will be included in each analysis (eg, all randomised participants), and in which group | Substudy: Statistical analysis. Parent protocol p. 9, Data analysis: intention-to-treat; randomised subjects analysed by randomised treatment. |
| 27c | How missing data will be handled in the analysis | Parent protocol p. 9, Data analysis: multiple imputation for missing data; dropout summarised by group and reason. Substudy adopts parent analytical principles. |
| 27d | Methods for any additional analyses (eg, subgroup and sensitivity analyses) | Substudy: Statistical analysis: exploratory descriptive analyses. Parent protocol p. 9, Data analysis: descriptive analyses specified; subgroup and sensitivity methods not explicitly specified. |
| 28a | Composition of data monitoring committee (DMC); summary of its role and reporting structure; statement of whether it is independent from the sponsor and funder; conflicts of interest and reference to where further details about its charter can be found, if not in the protocol. Alternatively, an explanation of why a DMC is not needed | Parent protocol p. 10, Phase VII: DMC consists of project management group (JPK, SB, ABA) and six coordinators; daily contact, weekly project meetings, and written reports to steering committee meeting monthly. Independence and separate DMC conflicts/charter not explicitly specified. |
| 28b | Explanation of any interim analyses and stopping guidelines, including who will have access to these interim results and make the final decision to terminate the trial | Parent protocol p. 9, Data analysis: no interim analyses; blinded reassessment of SD, correlation and ICC after six-month data for 50% of planned sample, with possible sample-size adaptation. Termination authority/stopping guidelines not specified. |
| 29 | Frequency and procedures for monitoring trial conduct. If there is no monitoring, give explanation | Parent protocol p. 10, Phase VII: daily contact during intervention, weekly project management meetings, written reports and monthly steering committee meetings (more often if indicated). |
| 30 | Plans for seeking research ethics committee/institutional review board approval | Substudy: Ethics approval and consent to participate Swedish Ethical Review Authority approval Dnr. 2025-03612-01 stated. |
| 31 | Plans for communicating important protocol modifications to relevant parties | Parent protocol pp. 11–12, Project organization: sponsors/funders notified, revised protocol sent to Investigator Site File, deviations documented with breach report form, and registry updated. |
| 32a | Who will obtain informed consent or assent from potential trial participants or authorised proxies, and how | Substudy: Ethics approval and consent to participate and Participants and eligibility criteria. Parent protocol p. 8, Phase VI: six coordinators collect consent forms before intervention. Substudy specifies oral/written consent and ethics-approved close-relative approval where capacity is lacking. |
| 32b | Additional consent provisions for collection and use of participant data and biological specimens in ancillary studies, if applicable | Parent protocol p. 11, Data availability: participants permit study use of data; additional ancillary-study consent not explicitly specified. No biological specimens described. |
| 33 | How personal information about potential and enrolled participants will be collected, shared, and maintained in order to protect confidentiality before, during, and after the trial | Substudy: Ethics approval and consent to participate and Availability of data and materials. Parent protocol p. 11, Data availability: data use limited to study purposes under GDPR. Detailed collection/sharing/access/retention safeguards not specified. |
| 34 | Provisions, if any, for ancillary and post-trial care, and for compensation to those who suffer harm from trial participation | Parent protocol p. 9, Safety: no anticipated harm and compensation for participation; post-trial care through standard dental remuneration programme. Specific arrangements for compensation for trial-related harm are not clearly described. |

Parent protocol: Persson Kylén J, Björns S, Hägglin C, et al. Evaluation of collaborative oral health care planning between older adults and personnel from public dental care and municipal care organizations: a study protocol for a cluster-randomized controlled study in Sweden. Trials. 2025;26:57. doi:10.1186/s13063-025-08753-6.
